# Inhibition of Enterovirus 71 Replication by 7-Hydroxyflavone and Diisopropyl-Flavon7-yl Phosphate

**DOI:** 10.1371/journal.pone.0092565

**Published:** 2014-03-24

**Authors:** Jianmin Wang, Haoxiang Su, Ting Zhang, Jiang Du, Sheng Cui, Fan Yang, Qi Jin

**Affiliations:** MOH Key Laboratory of Systems Biology of Pathogens, Institute of Pathogen Biology, Chinese Academy of Medical Sciences and Peking Union Medical College, Beijing, People's Republic of China; University of British Columbia, Canada

## Abstract

Enterovirus 71 (EV71) is the major causative agent of hand, foot, and mouth disease, which has been continuously prevalent in Asia in recent years. In children, severe cases can lead to death, and no prophylactic or therapeutic measures against EV71 infection are available. The 3C proteases of EV71 play an important role in viral replication and are an ideal drug target. In previous work, we resolved the crystal structure for EV71 3C^pro^. In this report, we took advantage of the automated docking program AutoDock 4.0 to simulate EV71 3C^pro^-ligand conformation. 7-hydroxyflavone (HF) and its phosphate ester(FIP) were predicted to bind with EV71 3C^pro^.In an in vitro protease inhibition assay, FIP inhibited EV71 3C^pro^ protease activity. Both flavones were highly active against EV71, protecting cells from EV71 infection. Replication of viral RNA and formation of EV71 plaque were all strongly inhibited in cells. These results indicated that HF and FIP may serve as potential protective agents in the treatment of patients with chronic EV71 infection.

## Introduction

Hand-foot-and-mouth disease (HFMD) is a common viral illness of infants and children that causes fever, sore throat, blisters, and skin rash for which there is no clinically effective therapy. HFMD disease outbreaks have occurred worldwide in recent years, causing devastating losses both economically and politically. EV71 was first isolated in California, USA, from patients with central nervous system diseases, and was eventually identified as one of the major causative agents for HFMD [1]. However, EV71 infection may also lead to severe neurologic diseases including meningitis, poliomyelitis-like disease, and fatal cases of encephalitis [2]. Recently, EV71 has been implicated in an increasing number of outbreaks throughout the world and is now classified as an emerging infectious disease with pandemic potential [3], [4], [5]. This virus is classified as a member of the enterovirus species A within the genus Enterovirus of the Picornaviridae family. It has a ∼7.4 kb positive-sense, single-stranded RNA genome with a single open reading frame encoding a polyprotein flanked by 5′- and 3′-untranslated regions. The coding region of the viral genome contains non-structural and structural viral proteins divided into three primary precursor molecules (P1, P2, and P3). The four structural proteins, VP1–4, are derived from the P1 portion of the polyprotein and form a structural protomer. Ultimately, 60 of these protomers form a non-enveloped icosahedral capsid to generate a mature virion [6], [7]. The non-structural proteins are found in the P2 and P3 regions.

Viral proteases 2A^pro^, 3C^pro^, and 3CD^pro^ are responsible for cleavage of the entire polyprotein to produce about 10 final products and a number of cleavage intermediates [8], [9], [10], [11], [12]. Because of the limited coding capacity of picornavirus genomes, precursor polyproteins and mature cleavage products actively participate in viral processes. The viral 3C is a multifunctional protein involved in binding with viral RNA and in RNA replication as well as a number of other biological processes. The 3C protein can trigger apoptosis through the caspase pathway in neuronal cells [13]. It has also been reported that 3C^pro^ can impair host RNA processing and polyadenylation by cleavage of the cellular CstF-64 protein, which subsequently enhances viral RNA replication [14]. In earlier work, we demonstrated that EV71 3C^pro^ can inhibit cellular antiviral responses of the infected cell by disruptions of the retinoic acid-inducible gene I, Toll-like receptor 3, and interferon regulatory factor 7 signaling pathways [15], [16], . In our work, we also resolved the structure for EV71 3C^pro^ [10], showing that the crystal structure of the unliganded EV71 3C^pro^ shares structural similarity with 3C proteases from hepatitis A virus, foot-and-mouth-disease virus, human rhinovirus (HRV), coxsackie B virus (CBV) and poliovirus [18], [19], [20], [21], [22]. Thus, the 3C^pro^ generally is considered as an appealing potential target for antiviral drug design. The 3C protease inhibitor rupintrivir inhibits in vitro replication of both HRV and EV71 by interfering with protease activity [23], [24].

In this study, we took advantage of the automated docking program AutoDock 4.0 to simulate EV71 3C^pro^-ligand conformation. We found that 7-hydroxyflavone (HF) from the flavone subgroup of flavonoids can be stably inserted into the pocket of EV71 3C^pro^. Our previous studies showed that phosphorylated flavonoids possess relatively stronger binding affinities towards proteins such as myoglobin, insulin, and lysozyme and more easily form non-covalent compounds with them, compared to non-phosphorylated forms [25], [26]. Moreover, phosphorylated flavonoids did exhibit stronger activity against HeLa tumor cells *in vitro* than non-phosphorylated flavonoids, in which these positive biomedical effects are mostly attributed to the potential of flavonoids to act as esters of phosphoric acid [27]. The phosphate ester (FIP, C_21_H_19_O_6_P) for HF was then generated. Further investigation revealed that FIP inhibited the protease activity of EV71 3C^pro^ in vitro. The MTS assay detected no obvious cytotoxicity for these two flavone compounds. In anti-EV71 assays, both flavones reduced the cytopathic effect (CPE) on EV71-infected rhabdomyosarcoma (RD) cells, and both HF and FIP suppressed EV71 replication in infected RD cells.

## Results

### HF binding with EV71 3C^pro^ in docking simulation

**Figure 1:**
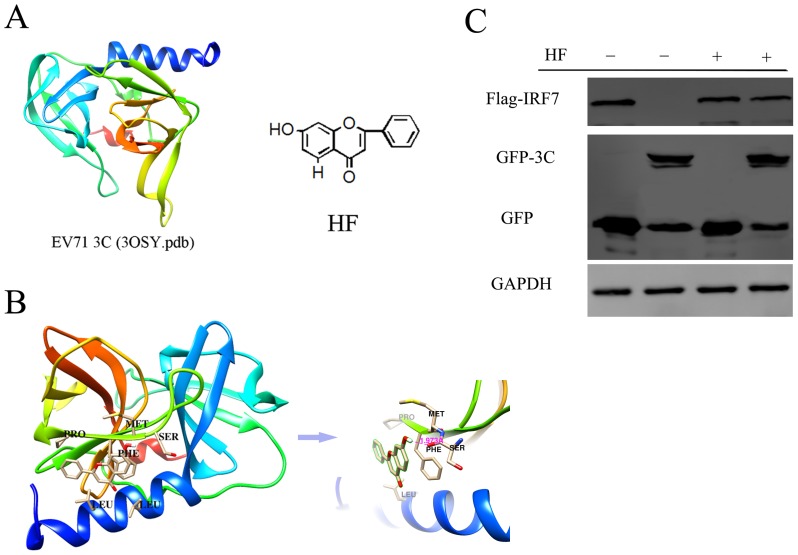
Molecular docking model for HF with viral 3C^pro^. (A) The chemical structure for EV71 3C protein and HF. (B)Top-ranked docking conformation for HF with viral 3C^pro^.

The structure for EV71 3C was shown in Figure 1A. To screen for 3C inhibitors, ligand molecules were docked into the EV71 3C protein using AutoDock 4.0 program, including myricetin, daidzin, kaempferol, rutin and HF. The stable conformation was formed between 3C and HF from the flavone subgroup. The structure for HF is shown in Figure 1A. As shown in Figure 1B, HF was predicted to bind with several hydrophobic and polar residues for EV71 3C^pro^, including LEU4, LEU8, SER111, MET112, PHE113, and PRO115. A hydrogen bond was formed between 7-OH of HF and SER111 around 2 Å. The corresponding BE was −5.89 kcal/mol, indicating the stability of the 3C^pro^-HF complex. We also tried to perform an in vitro protease inhibition assay to test the potential inhibitory effect against EV71 3C^pro^ for HF; however, HF was self-fluorescent at 538 nm with excitation at 355 nm in its neutral base form (data not shown). Our lab previously found that EV71 3C protein led to cleavage of human interferon regulatory factor 7 (IRF7) in co-transfected 293T cells [17]. To determine whether HF could suppress protease activity for viral 3C^pro^ in cells, we co-transfected 293T cells with viral 3C and human IRF7 expressing plasmids in the presence or absence of 100 μM HF. 24 hours later, cells were harvested and the cleavage of IRF7 was detected by western blot. As shown in Figure 1C, the cleavage of IRF7 by EV71 3C^pro^ was noticeably inhibited by HF.

### FIP inhibition of EV71 3C^pro^ protease activity in vitro

**Figure 2:**
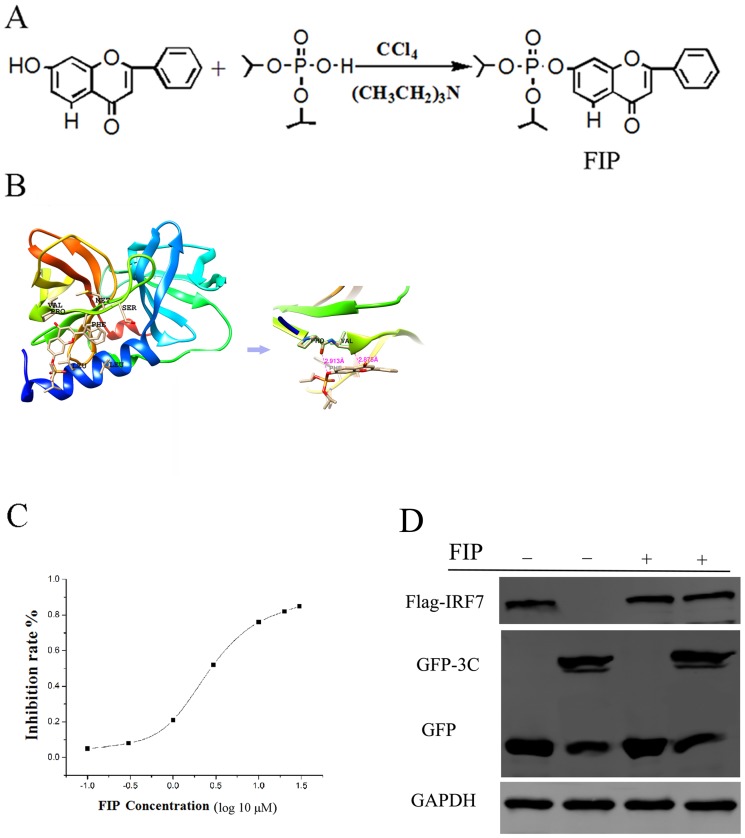
Inhibition of EV71 3C^pro^ protease activity by FIP in vitro. (A) Synthesis of FIP from HF by a simplified Atherton-Todd reaction. (B) Molecular docking model for FIP with viral 3C^pro^. (C) Protease activity for EV71 3C^pro^ was noticeably inhibited by FIP (*P*<0.05). Standard deviations of three independent experiments are shown.

FIP, the phosphate ester for HF, was generated by a simplified Atherton-Todd reaction (Figure 2A). A diisopropyl phosphate group was covalently anchored to 7-OH of HF. The structure for FIP was confirmed by NMR. The 3C^pro^-FIP conformation was also simulated by using the AutoDock 4.0 program. As illustrated in Figure 2B, FIP could also be inserted into the pocket of viral 3C^pro^ with the same binding sites as HF. With the presence of the diisopropyl phosphate group, FIP was predicted to form two H-bond with PRO115 and VAL116 through the P-O bond around 3 Å. The BE for the 3C^pro^-FIP complexes was −6.92 kcal/mol, which was even lower than that for 3C^pro^-HF complexes, suggesting a more stable incorporation. To test whether FIP could inhibit protease activity for EV71 3C^pro^, a protease inhibition assay was conducted. As shown in Figure 2C, FIP substantially inhibited the 3C^pro^ protease activity in vitro, with an IC_50_ of 2.48 μM. To further determine whether FIP could also suppress protease activity for viral 3C^pro^ in cells, we co-transfected 293T cells with viral 3C and human IRF7 expressing plasmids in the presence or absence of 100 μM FIP. After 24 hours, cells were harvested and the cleavage of IRF7 was detected by western blot. As shown in Figure 2D, the cleavage of IRF7 by EV71 3C^pro^ was noticeably inhibited by FIP.

### Cellular toxicity of HF and FIP in RD cells

**Figure 3:**
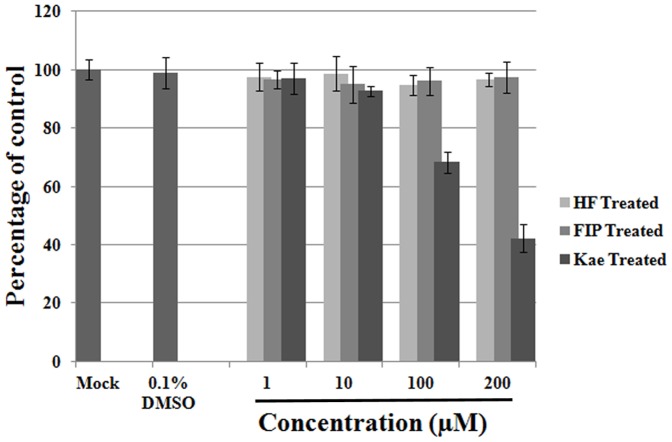
Cytotoxicity test for HF and FIP on RD cells. 1–200 μM HF and FIP showed no obvious inhibitory effect on RD cell proliferation (*P*>0.5). The mean value was obtained from four replicate wells, and means ± SD are shown.

Cellular toxicity of HF and FIP was assessed in RD cells using the MTS reduction assay, before application in the antiviral assay. When confluent monolayers were exposed to HF or FIP at concentrations of 1–200 μM for 48 h at 37°C, neither agent showed noticeable growth inhibitory effects compared to negative controls (Figure 3). Kae, a polyhydric flavonol, was also used as a positive control. For comparison, when 100 μM kae was added, proliferation of more than 30% of cells was inhibited (Figure 3).

### HF and FIP protection of RD cells from EV71 infection

**Figure 4:**
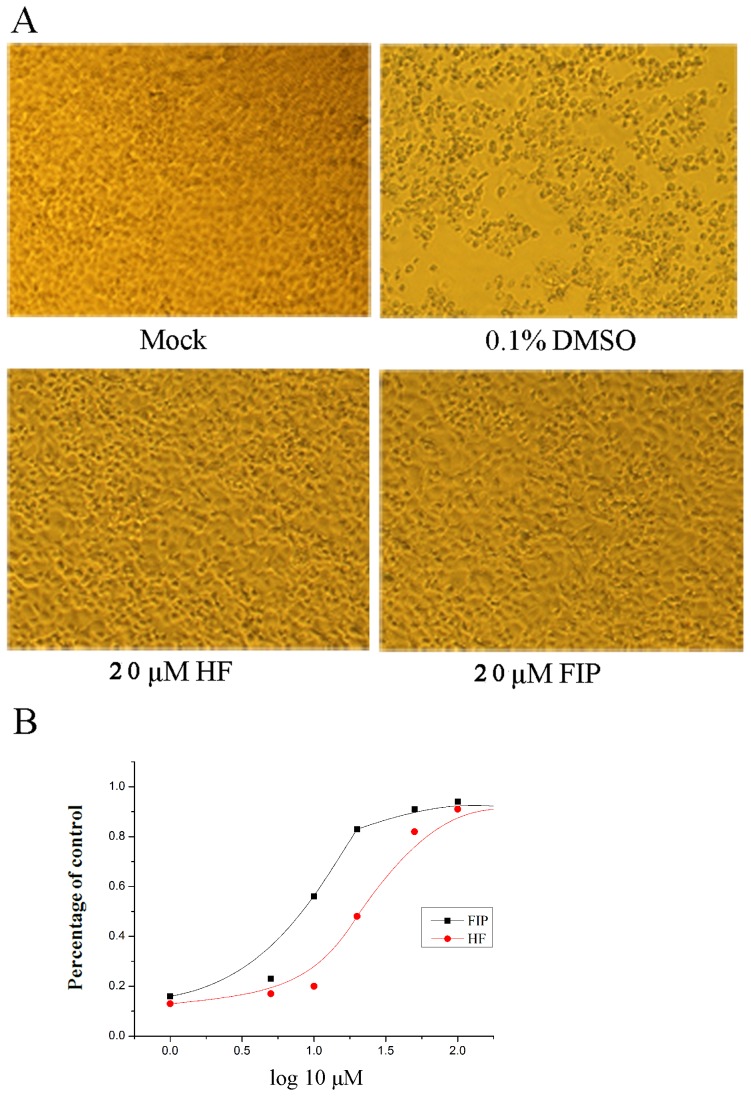
Effects of HF and FIP on EV71 infection. (A) Reduction of virus-induced cytopathic effects in RD cells by HF and FIP. (B) HF and FIP protected RD cells from EV71 infection (*P*<0.05). The experiment was performed in triplicate, and the bars represent means ± SD.

HF and FIP were then used in the anti-EV71 assay. To study the inhibition of EV71-induced CPEs by HF and FIP, RD cells were infected at 0.1 TCID_50_/cell; at the time of infection, 20 μM HF or FIP was added to the culture medium, respectively. In the negative control experiment, cells were exposed to 0.1% DMSO, which was used to dissolve the flavones. Morphological changes in the infected cells were examined by phase-contrast microscopy at 48 hpi. In contrast with the negative control, microscopy revealed that the cytopathic effects of EV71 on the RD cells were noticeably inhibited by HF and FIP at a concentration of 20 μM (Figure 4A). EC_50_ value was also estimated by testing cell viability at 72 hpi through MTS assay. As shown in Figure 4B, HF blocked EV71 proliferation with an EC_50_ of 19.95 μM, compared to 9.87 μM for FIP.

### HF and FIP inhibition of EV71 replication in cells

**Figure 5:**
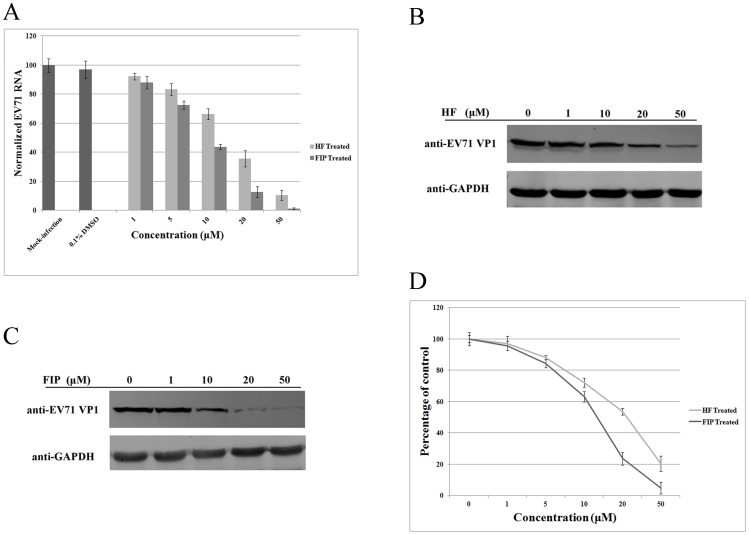
Inhibition of EV71 replication by HF and FIP in RD cells. (A) HF and FIP inhibited EV71 RNA accumulation (*P*<0.05). (B), (C) HF and FIP inhibited EV71 capsid protein VP1 synthesis. (D) EV71 plaque formation was reduced by addition of HF and FIP (*P*<0.05). The experiment was performed in triplicate, and the bars represent means ± SD.

To further study the inhibitory effect of HF and FIP on EV71, RD cells were infected at 0.1 TCID_50_/cell, and the inhibitory effect on EV71 RNA synthesis was measured at concentrations of 1, 10, 20 and 50 μM. EV71 RNA accumulation in infected RD cells decreased as the concentration of HF or FIP increased (Figure 5A). We found that 20 μM HF or FIP inhibited EV71 replication by more than 50%, and very low copies of EV71 were detected following addition of 50 μM flavones. HF and FIP inhibited EV71 RNA synthesis in a dose-dependent manner. Synthesis of viral proteins and infectious virions was also tested in the presence of HF or FIP. We infected RD cells with EV71 at 0.1 TCID_50_/cell in a series of concentrations of HF or FIP. Total cellular protein, including viral proteins, was collected 24 hpi. Western blotting was conducted to assess the amount of the capsid protein VP1 accumulated in infected RD cells relative to expression of cellular GAPDH as the internal control. Synthesis of viral VP1 protein in EV71-infected cells was inhibited by each flavone in a dose-dependent manner (Figure 5B and 5C). When the concentration of HF or FIP in the culture medium reached 20 μM, VP1 expression was dramatically reduced. In addition, both flaveones inhibited EV71 plaque formation in a dose-dependent manner, with an IC_50_ of 23.45 μM for HF and 13.63 μM for FIP, respectively (Figure 5D).

### HF and FIP inhibit replication of CAV and CBV

**Figure 6:**
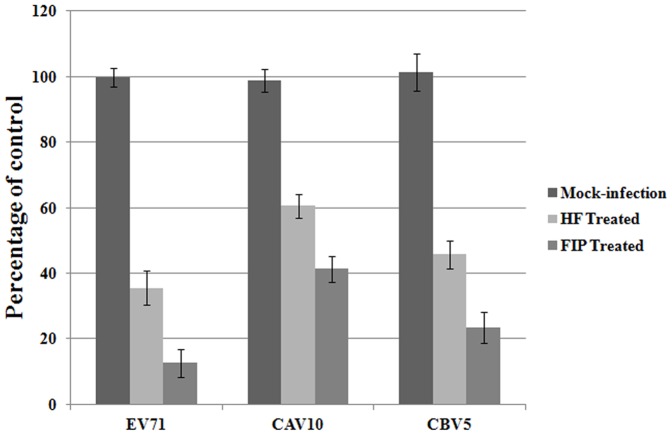
CAV10 and CBV5 replication is inhibited by HF and FIP. Concentrations of 20 μM HF and FIP substantially inhibited replication for both CAV10 and CBV5 (P<0.05). Standard deviations of three independent experiments are shown.

Since that the 3C^pro^ shares structural similarity among species of enteroviruses, we reasoned antiviral activity for HF and FIP against other enteroviruses. The effectiveness of HF and FIP against CBV5 and CAV10, was determined in this test. RD cells were infected with 0.1 TCID_50_/cell and treated with 20 μM HF and FIP. To measure the inhibitory effect of HF and FIP on the replication of CBV5 and CAV10, RT-PCR and relative quantitative real-time PCR were performed at 24 hpi. Cellular GAPDH was assayed as internal control. As shown in Figure 6, replication for both CBV5 and CAV10 was substantially inhibited by HF and FIP, with inhibition of CBV5 more than 50%, nearly the efficacy of the flavones against EV71.

Overall, these observations demonstrated that suppression of protease activity of viral 3C^pro^ by HF and FIP limited the infection of EV71, as well as CAV10 and CBV5, in cells.

## Discussion

EV71 infection continuously causes enormous losses all over the world [28], [29], [30], [31], [32], but no effective vaccines or therapeutic measures are available. Clinical infections are usually treated with intravenous immunoglobulin, with limited therapeutic efficacy [33]. An effective antiviral therapy against EV71 infection is urgently needed.

Plant-derived flavonoids are a large group of naturally occurring phenylchromones found in fruits, vegetables, tea, soy foods, and herbs. The structure of a flavonoid is usually characterized by a C6-C3-C6 carbon skeleton [34]. Numerous flavonoids may possess potential therapeutic benefits in a variety of viral infections. Baicalein, a member of the flavone subgroup of flavonoid, has been reported to inhibit replication of a number of viruses, including herpes simplex viruses 1 and 2 (HSV-1 and HSV-2) and Japanese encephalitis virus (JEV) [35], [36]. Quercetin, a member of the flavonol subgroup of flavonoids, is capable of inhibiting influenza virus, dengue virus, and JEV [36], [37], [38]. The antiviral activities of kaempferol and daidzin against JEV were also reported by our lab [39]. But to the best of our knowledge, no study has investigated the antiviral properties of HF and FIP from the flavone subgroup in vivo or in vitro. Here, we report in an antiviral assay, both HF and FIP were highly effective against infectious EV71 replication in cell cultures, even at relatively low concentrations and without obvious cytotoxicity.

The main protease for enteroviruses is 3C^pro^, which is responsible for the cleavage of eight junction sites within the viral polyprotein. Structural studies have revealed that the 3C proteins belong to a cysteine protease family. High-resolution crystal structures of the enzyme have also been resolved and used for the design of 3C protease inhibitors. Rupintrivir was initially designed as a peptidomimetic inhibitor to target HRV 3C^pro^ [24] but exhibits broad spectrum antiviral activity against enteroviruses, presumably because of the structural similarity of 3C^pro^ among the Picornaviridae family [21], [40], [41]. The 3C^pro^-rupintrivir has been resolved by our lab previously, and we traced the inhibitory effect of rupintrivir against EV71 3C^pro^ to the conformational changes arising from the association with inhibitor binding [23]. Currently, automated docking is extensively used as an effective means of quickly and accurately predicting biomolecular conformations and binding energies of protein-ligand complexes in molecular design [42], [43]. In this work, we took advantage of the automated docking program AutoDock 4.0 to simulate 3C^pro^-ligand conformation and found that HF and its phosphate ester FIP could bind with EV71 3C^pro^ in the docking simulation. The predicted binding sites were LEU4, LEU8, SER111, MET112, PHE113, and PRO115. As expected, the 3C^pro^-FIP complex was more stable in this study, as indicated by the negative BE value. The crystal structure showed that EV71 3C^pro^ had a typical chymotrypsin-like fold which was common in picornaviral 3C^pro^ [10]. GLU71 is essential for protease activity. An important surface loop, denoted as β-ribbon (123–133 aa), also plays an important role in recognizing the substrates. The KFRDI (82–86 aa) and VGK motifs were characterized as RNA binding motifs. According to the docking simulation, however, neither HF nor FIP recognized these conserved structures. We also used FIP for an in vitro protease activity inhibition assay in which it showed a potent inhibitory effect against EV71 3C^pro^ while excessive fluorogenic peptides was used. It was in consistent with the docking stimulation that binding sites for FIP do not occlude the peptide-binding site. It was plausible that the inhibitory effect of HF/FIP against EV71 3C^pro^ was also induced by the conformational changes arising from the association with inhibitor binding. In an antiviral test, both HF and FIP could suppress EV71 proliferation in infected RD cells, and synthesis of viral RNA and capsid protein were substantially reduced. CPE was also inhibited at low concentration when no cytotoxicity was detected. The antiviral activity for HF and FIP against CAV10 and CBV5 was also tested here. Replication for both viruses, especially for CBV5, was inhibited by the two flavones. These findings are consistent with previous results that the 3C^pro^ shares structural similarity among enteroviruses.

In conclusion, we demonstrated that HF and FIP exert a strong inhibitory effect on EV71 replication. This finding is particularly important because no effective antiviral drug is currently available for the prevention, treatment, and control of potentially fatal EV71 infections in humans.

## Materials and Methods

### Test compounds

HF (7-Hydroxy-flavone, C_15_H_10_O_3_, Mr: 238.24) and kaempferol (Kae, C_15_H_10_O_6_, Mr.286.23) were purchased from Sigma (St. Louis, MO, USA). FIP (diisopropyl-flavon7-yl phosphate, C_21_H_19_O_6_P) was synthesized by a simplified Atherton-Todd reaction. The new compound FIP was characterized by detailed spectroscopic analysis for C_21_H_19_O_6_P (MW:402): ^1^H NMR (400 MHz, dimethyl sulfoxide (DMSO)) δ 8.244–8.222 (m, 1 H), 7.944–7.925 (m, 2 H), 7.563–7.511 (m, 2 H), 7.298–7.272 (m, 2 H), 6.838 (s, 1 H), 4.832 (q, *J* = 6.4 Hz, 2 H), and 1.401 ppm (dd, *J* = 16.8, 6.0 Hz, 12 H); ^31^P NMR (400 MHz, D_2_O), d: −8.772; ESI-MS/MS, m/z 403 [M+H]^+^. For cell experiments, the compounds were dissolved in DMSO and finally diluted in culture medium. To avoid toxicity or interference from the solvent, the maximum concentration of DMSO in the cell test medium was lower than 0.1%.

### Cell culture

RD cells (ATCC, USA) were propagated and maintained in minimum essential medium (HyClone, Logan, UT, USA) supplemented with 10% fetal bovine serum (Invitrogen, Carlsbad, CA, USA) and 100 U/ml penicillin, and 100 μg/ml streptomycin at 37°C under 5% CO_2_. Cell numbers and proliferation were determined by direct cell number counting using CountStar (Inno-Alliance Biotech, Beijing, China) after staining with trypan blue.

### Virus infection and drug treatment

The EV71 strain (Shzh-98, GenBank accession no. AF302996) was used. Coxsackie A viruses (CAV10) and CBV5 were isolated, sequenced and stored in our lab. Viruses were propagated in RD cells. HF and FIP were added to cell cultures in a concentration series at the time of infection or at the indicated time post-infection. Western blotting and quantitative real-time PCR were performed at indicated time post-infection as described below. Virus titer was determined as TCID_50_ on RD cells by the Reed-Muench method [44]. Representative results are shown.

### Plaque reduction assay

RD cells were seeded into 6-well plates at a density of 10^6^ cells/well and incubated for 24 h to form a monolayer. The cells were then inoculated with EV71 at 100 PFU/well and mixed with or without the serially diluted testing compounds. After incubation at 37°C for 1 h, the culture medium was replaced with overlay medium containing 1.5% agar with the testing compounds at the corresponding concentrations and incubated in a CO_2_ incubator for 3 days at 37°C until plaques appeared. The cells were then fixed with 10% formaldehyde and stained with 0.05% crystal violet. The 50% inhibitory concentration for each flavone compound was calculated using the Forecast function of Microsoft Excel.

### MTS assay

Before the cell experiments, cellular toxicity for the tested compounds was tested according to a previously reported cell viability assay [45]. The cells were harvested in the log phase of growth and inoculated onto 96-well plates at a final concentration of 3 × 10^3^ cells per well. After 24 h of incubation at 37°C under 5% CO_2_, the cell cultures were treated with the flavones at 1, 10, 100 and 200 μM concentrations, respectively. [3-(4,5-dimethylthiazol-2-yl)-5-(3-carboxymethoxyphenyl)-2(4-sulfophenyl)-2H-tetrazolium/phenazine methosulfate (MTS/PMS; Promega, Madison, WI, USA) 20 μl was added to each well, and the absorbance at 490 nm was measured according to the manufacturer's recommendations. The mean value was obtained from four replicate wells. A control group treated with 0.1% DMSO was performed simultaneously.

### Molecular docking simulation in EV71 3C^pro^ inhibition

For estimating the potential interaction and the conformation of the 3C^pro^–ligand complex, the ligands were docked into the EV71 3C protein using the AutoDock 4.0 program (Scripps Research Institute). The 3D structures for the ligands were constructed and minimized using Chemsketch 3.5 and Omega 2.0 software (OpenEye Scientific Software, USA). For docking studies, the crystal structure of the EV71 3C^pro^ (PDB no. 3OSY) was used. The docking simulation was conducted with a rigid construction for viral 3C protein, while the structures for the ligands were allowed to vary. The predicted protein-ligand complexes were optimized and ranked according to the empirical scoring function, ScreenScore, which estimated the binding free energy (BE) of the ligand-receptor complex. The docking of the 3C^pro^-ligand molecule was successful, as indicated by statistically significant scores. Each docking was performed twice, and each operation screened 250 conformations for the 3C-ligand complex that could have been advantageous for docking; namely, each docking could have 500 preferred conformations. The binding energy and the frequency of the top 5 docking results for 3C-HF/FIP complex were provided in Table 1. The most stable conformation as distinguished by the minimal binding energy was shown.

**Table 1:** Top-ranked docking conformation for HF and FIP with viral 3C^pro^.

	Frequency (%)	Binding Energy (kcal/mol)
HF-1.B	63.6	−5.89
HF-S1.A	4.6	−4. 91
HF-S1.B	3.8	−4. 65
HF-S1.C	1.6	−4.09
HF-S1.D	23.4	−5.47
FIP-2.B	54.8	−6.92
FIP-S1.E	18.2	−6.07
FIP-S1.F	10.2	−4. 68
FIP-S1.G	8.6	−4. 36
FIP-S1.H	6.4	−4. 31

The predicted binding model for HF-1.B and FIP-2.B was shown in Figure 1B and 2B; HF-S1.A-D and FIP-S1.E-H were in turn shown in Figure S1.

### Protease inhibition assay

A protease inhibition assay was conducted as described previously [41] to evaluate the inhibitory effect of protease activity of EV71 3C^pro^ by each flavone compound. The fluorogenic peptide Dabcyl-RTATVQGPSLDFE-Edans (EV71 3C^pro^ cleaves the peptide bond between the underlined residues), corresponding to the EV71 polyprotein 3B/3C autoprocessing site, was used as the substrate. Recombinant EV71 3C protease was produced by prokaryotic expression as described previously [10] and used as the enzyme in the protease assays. Viral 3C^pro^ was first incubated with each flavone for 30 min at 4°C to allow sufficient binding. The reactions were then performed with 4 μM protease and 150 μM fluorogenic peptide in a buffer of Tris-HCl (pH 7.0), 200 mM NaCl, and 2 mM dithiothreitol at 30°C. The fluorescence change resulting from the reaction was monitored by using a fluorescence plate reader (Fluoroskan Ascent from ThermoLabsystems, Finland) at an excitation wavelength of 355 nm and by monitoring the emission at 538 nm and was then converted to substrate concentration. An enhanced fluorescence resulting from the cleavage of the peptide was detected.

### Reverse transcription-PCR (RT-PCR)

At 24 h post-infection (hpi), total cellular RNA and viral RNA were extracted from each well using the RNAeasy Mini kit (Qiagen, Hilden, Germany) according to the manufacturer's instructions. RT-PCR was conducted with the Superscript First-Strand Synthesis System (Invitrogen, Carlsbad, CA, USA) in a 20 μl reaction mixture with 1.2 μg total RNA and 50 ng random hexamers according to the manufacturer's instructions. cDNA samples were then subjected to quantitative real-time PCR detection as described below.

### Quantitative real-time PCR

Quantitative real-time PCR was conducted using an ABI Prism 7000 Real-time PCR System (Applied Biosystems, Carlsbad, CA, USA) and a Power SYBR Green PCR Master Kit (Invitrogen, Carlsbad, CA, USA). The region of nt 1–750 in the EV71 strain Shzh-98 genome sequence was amplified by PCR and cloned into a pGEM-T Easy vector (Promega, Fitchburg, WI, USA) to construct the standard plasmid. Using the standard plasmid, we obtained the standard curve. Reactions for quantitative real-time PCR contained 2 μl of cDNA, 1 μl of each primer, and 25 μl Power SYBR Green PCR Master Mix in a total volume of 50 μl. Primers used were as described previously [46]. The absolute quantity of viral RNA was calculated by using the standard curve. A melting curve analysis was performed to verify the authenticity of the amplification. For quantitative detection of CAV10 and CBV5, relative quantitative real-time PCR was performed as described previously [46]. To control for equal loading, cellular glyceraldehyde 3-phosphatase dehydrogenase (GAPDH) was also detected as an internal control.

### Western blot analysis

Cells were pelleted by centrifugation and lysed in buffer containing 100 mM NaCl, 20 mM Tris (pH 8.0), 0.5% NP-40, 0.25% sodium deoxycholate, and 1 mM EDTA with protease inhibitor cocktail (Roche, Indianapolis, IN, USA). Supernatant was collected at 15,000 g for 15 min. Aliquots of cell lysates were electrophoresed on 12% SDS-PAGE gels and transferred to nylon polyvinylidene difluoride membranes (PVDF, Hybond P, Piscataway, NJ, USA). The membranes were blocked with 5% nonfat dry milk, and proteins on the membrane were probed with primary antibodies as indicated at 4°C overnight followed by incubation with corresponding IRD Fluor 800–labeled IgG or IRD Fluor 680–labeled IgG secondary antibody (Li-Cor Inc., Lincoln, NE, USA) for 1 h at room temperature. After washing, the membranes were scanned with the Odyssey infrared imaging system (Li-Cor Inc., Lincoln, NE, USA) at a wavelength of 700 to 800 nm, and the molecular sizes of the developed proteins were determined by a comparison with prestained protein markers (Fermentas, Glen Burnie, MD, USA). The EV71 capsid protein VP1 was detected by anti-EV71 VP1 monoclonal antibody (eENZYME, Gaithersburg, MD, USA). GFP-3C and Flag-IRF7 was detected by using mouse anti-GFP and anti-Flag (Beyotime, Suzhou, China). To control for protein loading, levels of the housekeeping protein GAPDH were assessed using mouse anti-GAPDH (Beyotime, Suzhou, China).

### Statistical analysis

At least three independent experiments were carried out for each variable. Statistical analysis was performed using SSPS 10.0. Differences with *P*<0.05 were considered statistically significant. All results are presented as mean ± SD.

## Supporting Information

Figure S1 Molecular docking model for HF and FIP with viral 3C^pro^.
(TIF)
