# Supplementary figures and images for: Inhibition of Enterovirus 71 Replication by 7-Hydroxyflavone and Diisopropyl-Flavon7-yl Phosphate
Source: PLoS One. 2014 Mar 24;9(3):e92565. doi: 10.1371/journal.pone.0092565 (PMC3963929; doi:10.1371/journal.pone.0092565)

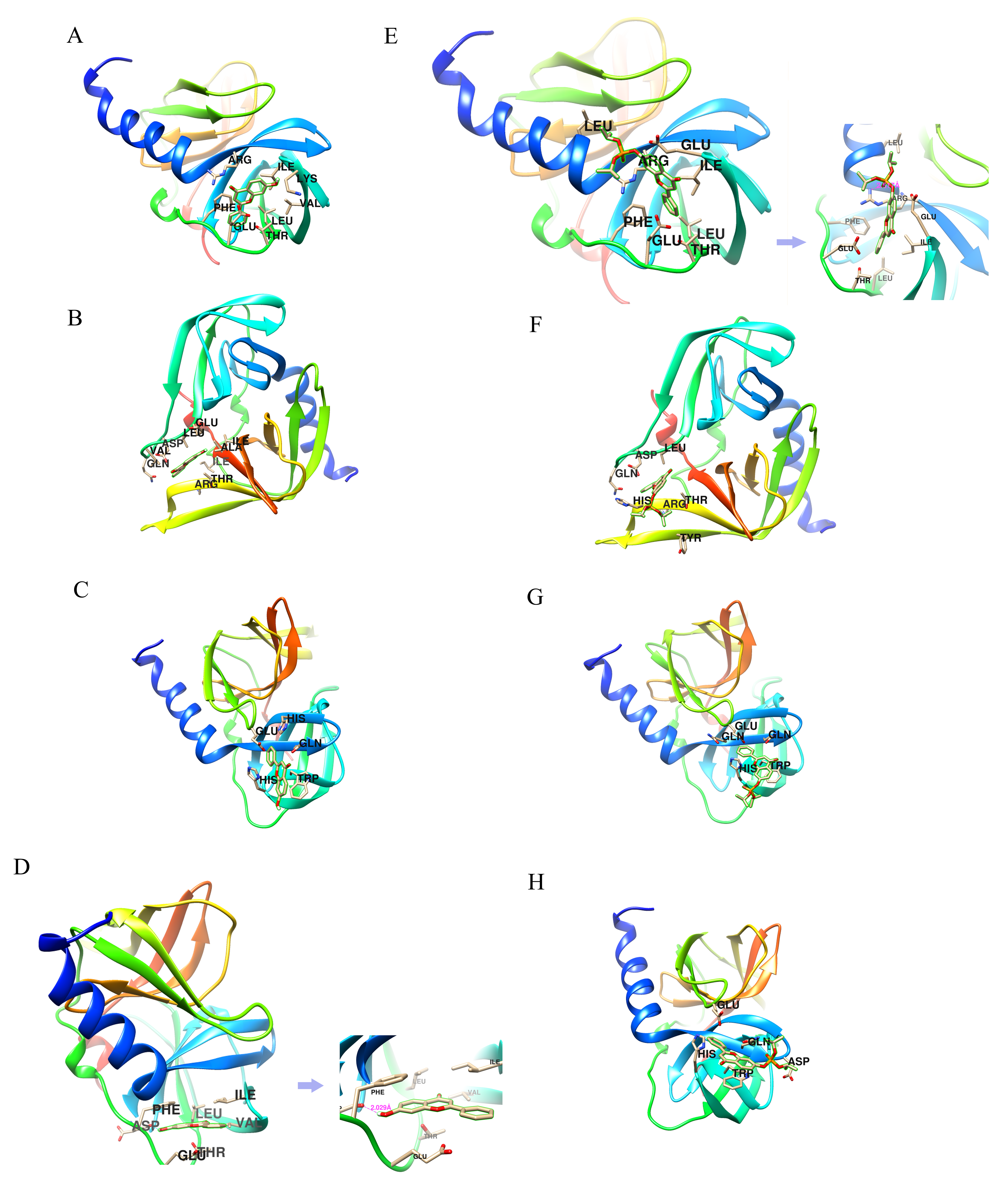

Supplement: Figure S1 — Molecular docking model for HF and FIP with viral 3Cpro. (TIF) [file pone.0092565.s001.tif]
